# Xiphodynia: A Case of Post-traumatic Sternal Pain Managed With Bipolar Pulsed Radiofrequency

**DOI:** 10.7759/cureus.85853

**Published:** 2025-06-12

**Authors:** André Aguiar, Noélia Carrillo-Alfonso, Afonso Eliseu, Ana Catarina Segundo, Ana Lares

**Affiliations:** 1 Anesthesiology, Unidade Local de Saúde do Algarve - Hospital de Faro, Faro, PRT

**Keywords:** bipolar pulsed radiofrequency, chronic pain management, non-cardiac chest pain, ultrasound-guided intervention, xiphodynia

## Abstract

Xiphodynia is a rare, under-recognized musculoskeletal pain syndrome often misdiagnosed due to its nonspecific symptoms. We report a case of a 32-year-old male with chronic sternal pain following trauma, refractory to multiple conservative treatments and local anesthetic infiltration. Clinical evaluation revealed focal tenderness over the xiphoid process, with pain reproduced on palpation and bilateral anterior thoracic radiation. A transient diagnostic infiltration with 2 mL of 1% lidocaine confirmed the xiphoid process as the source of pain. The patient underwent ultrasound-guided bipolar pulsed radiofrequency (pRF) targeting the xiphoid process, resulting in substantial and sustained pain relief. This case highlights the importance of including xiphodynia in the differential diagnosis of persistent chest pain and demonstrates that pRF may be an effective option for refractory cases.

## Introduction

Xiphodynia is a musculoskeletal pain syndrome characterized by localized or referred pain originating from the xiphoid process or its adjacent structures. Its nonspecific symptoms, often mimicking cardiac ischemia, gastroesophageal reflux, or costochondritis, frequently lead to misdiagnosis. Anatomical proximity to visceral organs and overlapping pain referral patterns contribute to under-recognition. Some studies suggest diagnostic delays averaging six to 12 months in refractory cases. Risk factors include prior trauma, repetitive mechanical strain, anatomical variations, and systemic inflammatory conditions [1-5].

The xiphoid process receives sensory innervation from the terminal branches of the T5-T9 intercostal nerves, which transmit nociceptive signals during mechanical or inflammatory irritation. Bipolar pulsed radiofrequency (pRF) modulates pain perception by delivering high-voltage, short-duration currents to these peripheral nerves, suppressing aberrant nociceptive signaling while avoiding thermal tissue damage [1,4,6].

Recent literature continues to affirm that xiphodynia is rare and underdiagnosed, with limited epidemiological data. While the classic 1955 Lipkin study estimated an incidence of about 2% in hospital populations [2], more recent case reports and small series suggest that xiphodynia may be more common than previously thought, particularly in middle-aged adults and possibly with a male predominance [6-8]. However, comprehensive, large-scale epidemiological studies are still lacking.

## Case presentation

A 32-year-old male, with no relevant past medical history, except for previously diagnosed and adequately treated syphilis, presented with persistent sternal pain beginning a few days after a motorcycle accident four years prior. The trauma involved a collision with a light vehicle; imaging at the time (head CT, chest X-ray, spinal X-ray) revealed no sternal or rib fractures. Subsequent evaluations, including ECG, stress testing, echocardiogram, sternal X-ray, and abdominal CT, were unremarkable.

The pain progressively worsened and was exacerbated by physical exertion and palpation of the central sternal region. The patient, previously active in bodybuilding, ceased training due to pain. On examination, focal tenderness was isolated to the xiphoid process, with bilateral anterior thoracic radiation upon deep palpation. There were no neuropathic descriptors except for a burning sensation. The average Numeric Rating Scale (NRS) pain score was 7-8/10.

He was initially managed with NSAIDs (naproxen), oral metamizole, and lidocaine patches without adequate relief. A periarticular infiltration with local anesthetic (2 mL of 1% lidocaine) and corticosteroid (dexamethasone) provided only temporary benefit. Upon referral to the pain unit, pregabalin was initiated (titrated to 50 mg BID), with mild tolerability issues.

Given the refractory nature of the pain and positive response to local anesthetic infiltration, the treating physician recommended ultrasound-guided bipolar pulsed radiofrequency (pRF).

The procedure was performed six weeks after the consultation. The patient was positioned supine with his arms by his sides. A high-frequency linear ultrasound probe was used to identify the xiphoid process along with adjacent anatomical landmarks (including the costal margins and the sternal body). After appropriate skin antisepsis and local anesthesia with 1% lidocaine at the needle entry sites, an in-plane transverse approach was adopted.

Two 22 G radiofrequency needles (10 cm in length with a 10 mm active tip) were advanced under real-time ultrasound guidance so that their tips lay on either side of the xiphoid process. The inter-electrode distance was maintained at less than 10 mm to ensure effective energy delivery. Sensory testing was then performed: a positive response was recorded at stimulation levels of less than 0.5 V, while motor stimulation remained negative up to 2 V. These parameters provided confirmation of accurate needle placement targeting the sensory branches of the lower intercostal nerves.

**Figure 1:**
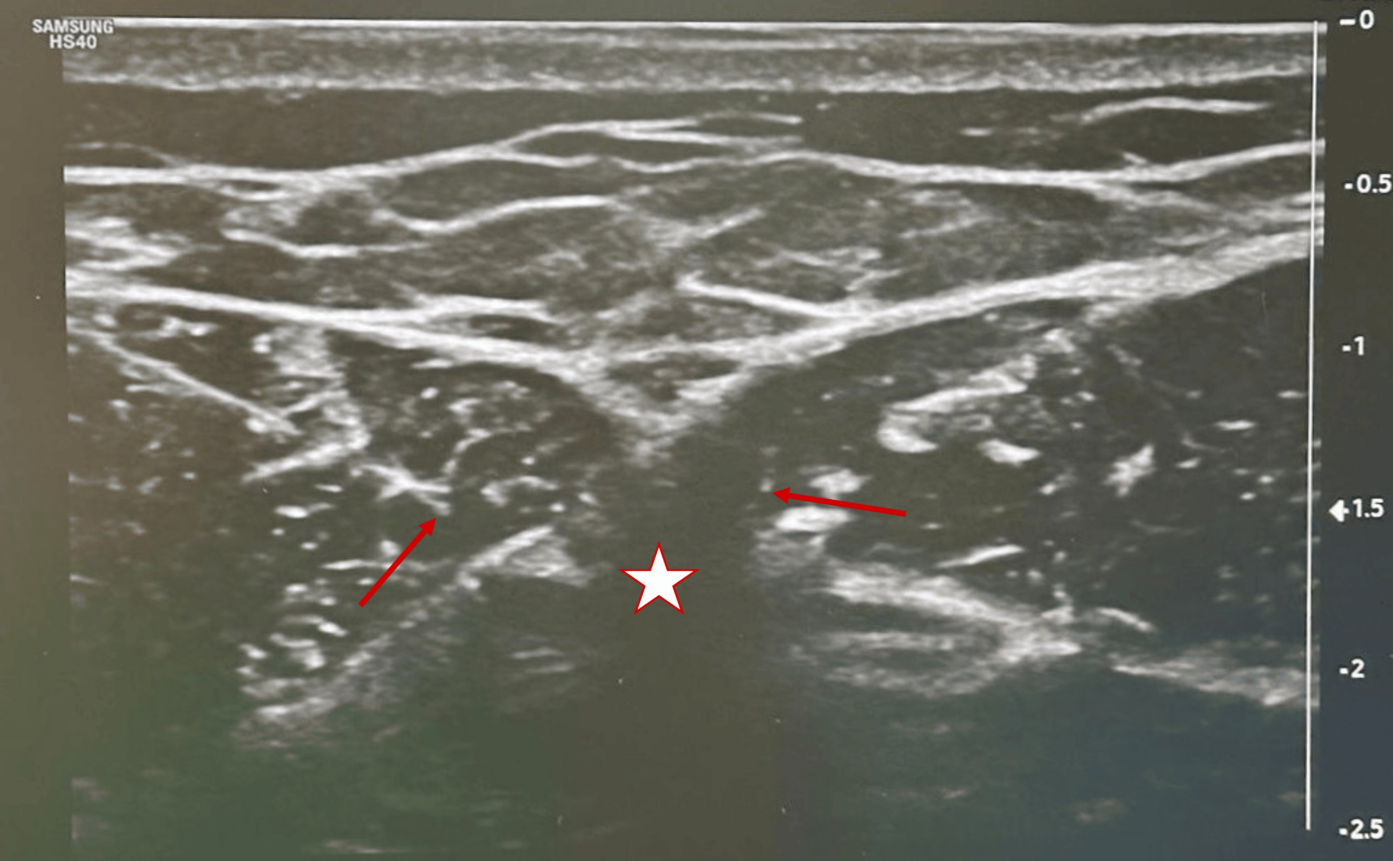
Red arrow: needle tip; star: shadow acoustic xiphoid appendix

Following successful sensory testing, bipolar pRF was administered with the following technical parameters: maximum temperature of 40°C, frequency of 2 Hz, voltage of 45 V, pulse duration of 20 ms, alternating polarity, in three cycles of two minutes (total six minutes) (Figure 1).

At the one-month follow-up, the patient experienced significant relief of baseline pain, with his average NRS dropping from 8/10 to 3/10. Despite the sustained improvement in average pain, peak pain levels remained high (8/10) during breakthrough episodes, although these episodes became less frequent. At the six-month reassessment, a slight increase in average pain to 5/10 was noted, particularly during nighttime and periods of stress. These breakthrough episodes responded to oral analgesics, and given the patient’s overall favorable response to pRF, a repeat intervention is being considered.

The evolution of the Numeric Rating Scale (NRS) scores is summarized in Table 1.

**Table 1:** Pain score evolution (Numeric Rating Scale (NRS))

Timepoint	Maximum NRS	Minimum NRS	Average NRS
Before the procedure	8/10	7/10	8/10
One month after the procedure	8/10	0/10	3/10
Six months after the procedure	8/10	0/10	5/10

## Discussion

Xiphodynia is an under-recognized and potentially debilitating musculoskeletal pain syndrome that originates from irritation or inflammation of the xiphoid process. This structure, located at the inferior aspect of the sternum, undergoes ossification in adulthood and serves as an attachment point for several anatomical structures, including the diaphragm, rectus abdominis, and transversus thoracis muscles [1]. Due to its central location and shared innervation pathways with thoracoabdominal structures, pain from the xiphoid process often mimics cardiac, gastrointestinal, or costosternal conditions, leading to frequent misdiagnosis and unnecessary investigations [1,2,3].

The xiphoid process is innervated primarily by the terminal branches of the T5-T9 intercostal nerves, which transmit nociceptive signals in response to inflammation, mechanical stress, or direct trauma. In our case, the pain pattern was localized and reproducible upon palpation, with bilateral radiation to the anterior thoracic wall, which did not follow a dermatomal or visceral distribution, consistent with xiphodynia. Diagnostic confirmation was supported by transient symptom relief following a local lidocaine injection, a maneuver considered both diagnostic and therapeutic [1,4].

Several predisposing factors have been identified, including previous chest trauma, repetitive mechanical overload (weightlifting), childbirth, and systemic inflammation (such as ankylosing spondylitis or rheumatoid arthritis) [1,4,5]. An increasingly recognized but underexplored contributing factor is anatomical variation of the xiphoid process, particularly its angulation. Anterior or ventral deflection, as well as elongation or abnormal ossification, may increase susceptibility to irritation through contact with soft tissues or repetitive mechanical strain during torso flexion or abdominal pressure [4,5,7,8]. 

The diagnosis of xiphodynia is primarily clinical, centered on reproducibility of pain on xiphoid palpation after exclusion of more serious etiologies such as acute coronary syndrome, gastroesophageal reflux, and biliary disease [2-4]. There is currently no validated clinical scoring system for xiphodynia; bedside palpation and diagnostic blocks remain the key tools. Imaging modalities, including ultrasound and CT scans, help rule out alternative diagnoses and, in select cases, reveal xiphoid hypermobility, calcifications, or anatomical anomalies. However, standardized imaging protocols and quantifiable diagnostic markers (xiphisternal angle measurements) are still lacking [4,5].

In terms of management, most patients respond to conservative therapies including NSAIDs, topical therapies, physical therapy, and postural modification. Local anesthetic or corticosteroid injections can provide both diagnostic and therapeutic benefits. In refractory cases, targeted interventional procedures may be indicated [4,6,9]. In this case, bipolar pulsed radiofrequency (pRF) was recommended based on persistent symptoms and prior positive response to injection. pRF exerts neuromodulatory effects by delivering high-voltage, short-duration currents to the targeted peripheral nerves, typically at temperatures below 42°C, avoiding tissue destruction while suppressing aberrant pain signaling [6,10].

When pRF fails or relief is short-lived, xiphoidectomy has shown promising results, with resolution rates of up to 89-100% in selected patients [9,10].

In this case, pRF resulted in a significant, sustained reduction in average pain, although peak pain episodes persisted, albeit with reduced frequency and impact. The literature suggests that pRF offers relief for several weeks to months, and repeat procedures are a viable option if symptoms recur [6,10]. Given the patient’s favorable initial response and partial return of symptoms, a repeat pRF procedure is currently being considered in the upcoming weeks. Predictors of failure in conservative management include persistent pain despite NSAIDs/injections, severe mechanical tenderness, symptom duration >6 months, and lack of sustained response to multimodal therapy [4,9].

However, direct comparative trials between conservative, interventional, and surgical strategies remain scarce, and no treatment guidelines have been established. The lack of prospective, high-quality studies also limits generalizability.

Future research should prioritize large-scale epidemiological assessments to establish true prevalence; imaging criteria (xiphisternal angle thresholds); comparative efficacy of conservative, interventional, and surgical modalities; and the development of a clinical staging system for xiphodynia based on chronicity, anatomical variation, and treatment response.

## Conclusions

Xiphodynia remains an under-recognized cause of anterior chest pain, often misdiagnosed due to its nonspecific and variable presentation. This case highlights the importance of considering xiphodynia in the differential diagnosis of persistent sternal pain, particularly following trauma. Diagnosis relies on clinical evaluation, with the reproduction of symptoms upon palpation being an essential component. In cases resistant to conservative therapies, interventional treatments such as bipolar pulsed radiofrequency can offer significant relief. Increased awareness and further research are essential to improve diagnostic accuracy and treatment protocols and optimize patient outcomes.
